# Supplementary material for: Multi-Probiotic Lactobacillus Supplementation Improves Liver Function and Reduces Cholesterol Levels in Jeju Native Pigs
Source: Animals (Basel). 2021 Aug 5;11(8):2309. doi: 10.3390/ani11082309 (PMC8388395; doi:10.3390/ani11082309)

Multi-probiotic *Lactobacillus* supplementation improves liver function and reduces cholesterol levels in Jeju native pigs

Dahye Kim, Yunhui Min, Jiwon Yang, Yunji Heo, Mangeun Kim, Chang-Gi Hur, Sang-Chul Lee, Hak-Kyo Lee, Ki-Duk Song, Jaeyoung Heo, Young-Ok Son, Dong-Sun Lee

Supplementary material

Supplementary Table S1. Composition, nutrient, and energy content of the basal diet.

	Basal diet
Ingredients, g/kg	
Corn	392.5
Wheat	200.0
Soybean meal	137.3
Wheat bran	104.0
Soybean hull	54.1
Rice bran	40.0
Fiber feed	20.0
Limestone	15.6
Tallow	10.0
Monodicalcium phosphate	9.0
NaCl	5.0
Choline chloride	2.2
Acidifier	2.0
Tryptophan	2.0
Lysine	1.8
Toxin-binder	0.5
Vitamin premix [a]	2.0
Mineral premix [b]	2.0
Total	1,000.0
Analyzed crude nutrient and energy contents	
Moisture, %	12.98
Crude protein, %	15.65
Crude fat, %	3.58
Crude ash, %	2.83
Crude fiber, %	5.64
Gross energy, Kcal/kg	3,836.00

[a]The vitamin premix provided per kg diet: vitamin A, 12,000 IU; vitamin D3, 2,000 IU; vitamin E, 100 IU; vitamin K3, 4.5 mg; vitamin B1, 2 mg; vitamin B2, 7 mg; vitamin B3, 45 mg; pantothenic acid, 30 mg; vitamin B6, 4.5 mg; Biotin, 0.5 mg; Folic acid, 3.5 mg; vitamin B12, 0.03 mg; antioxidant, 6.6 mg

[b]The mineral premix provided per kg diet: Fe, 150 mg; Zn, 85 mg; Mn, 37 mg; Cu, 11 mg; Co, 2 mg; 3 mg; Se, 0.15 mg

Supplementary Table S2. Primers used for RT-PCR or qRT-PCR

Primer name	Primer sequence (5′ to 3′)
pGAPDH F	ATCACTGCCACCCAGAAGAC
pGAPDH R	AGCCCCAGCATCAAAGGTAG
mAdiponectin F	GGA ACT TGT GCA GGT TGG AT
mAdiponectin R	CTT GCC AGT GCT GTT GTC AT
mChemerin F	TAC AGG TGG CTC TGG AGG AG
mChemerin R	GGC AAA CTG TCC AGG TAG GA
pVisfatin F	AGT TAC ATG ACT TTG GTT ACA
pVisfatin R	TGA TAT CCA CGC CAT CTC CTT G
pTLR4 F	GGC AGT GTG CTG AGG AGA GA
pTLR4 R	CGA GGA ATC CTG GCA TT
pNOD1 F	TTC GTC TTC GTG CTC CA
pNOD1 R	GCT CAC TGG ATC GGT GTT GA
pNOD2 F	TCT GCC TGG AGG AGA ACC AT
pNOD2 R	GAG GTG ACG TGG TTG GA
pMyD88 F	CTG CGT CTG GTC CAT TGC TA
pMyD88 R	TGG AGA GAG GCT GAG TGC AA
pIL-1B F	GTGTTCTGCATGAGCTTTGTG
pIL-1B R	GGCTTTCCTTAGGGAGAGAGG
pTNF-α F	CAGCTGGAGAAGGATGATCGA
pTNF-α R	CCAAAATAGACCTGCCCAGATT
pIL-6 F	GACCTGCTTGCTGAGAATCACC
pIL-6 R	ATCCACTCGTTCTGTGACTGC
pINF-γ F	GAGCCAAATTGTCTCCTTCTAC
pINF-γ R	CGAAGTCATTCAGTTTCCCAG
pMyoD F	GTCACCCAGGAGCACAAAT
pMyoD R	CCTGAGCAAAGTCAACGAG
pPax7 F	CCA TGG CTG TGT CTC CAA GA
pPax7 R	CGG ATG GAC CCG GTC TCT
pMyBPH F	AGTGCAGAAGGCAGACAAA
pMyBPH R	AAGACCCGGAAGGAGTAAGA
mPPARα F	TGCAAACTTGGACTTGAACG
mPPARα R	AATCCCCTCCTGCAACTTCT
mPPARβ F	TTGAGCCCAAGTTCGAGTTTGCTG
mPPARβ R	ATTCTAGAGCCCGCAGAATGGTGT
mPPARγ F	CTG GCC TCC CTG ATG AAT AA

mPPARγ R	CAC GTG CTC TGT GAC GAT CT

Direction: F means forward, and R means reverse

Supplement Figure S1

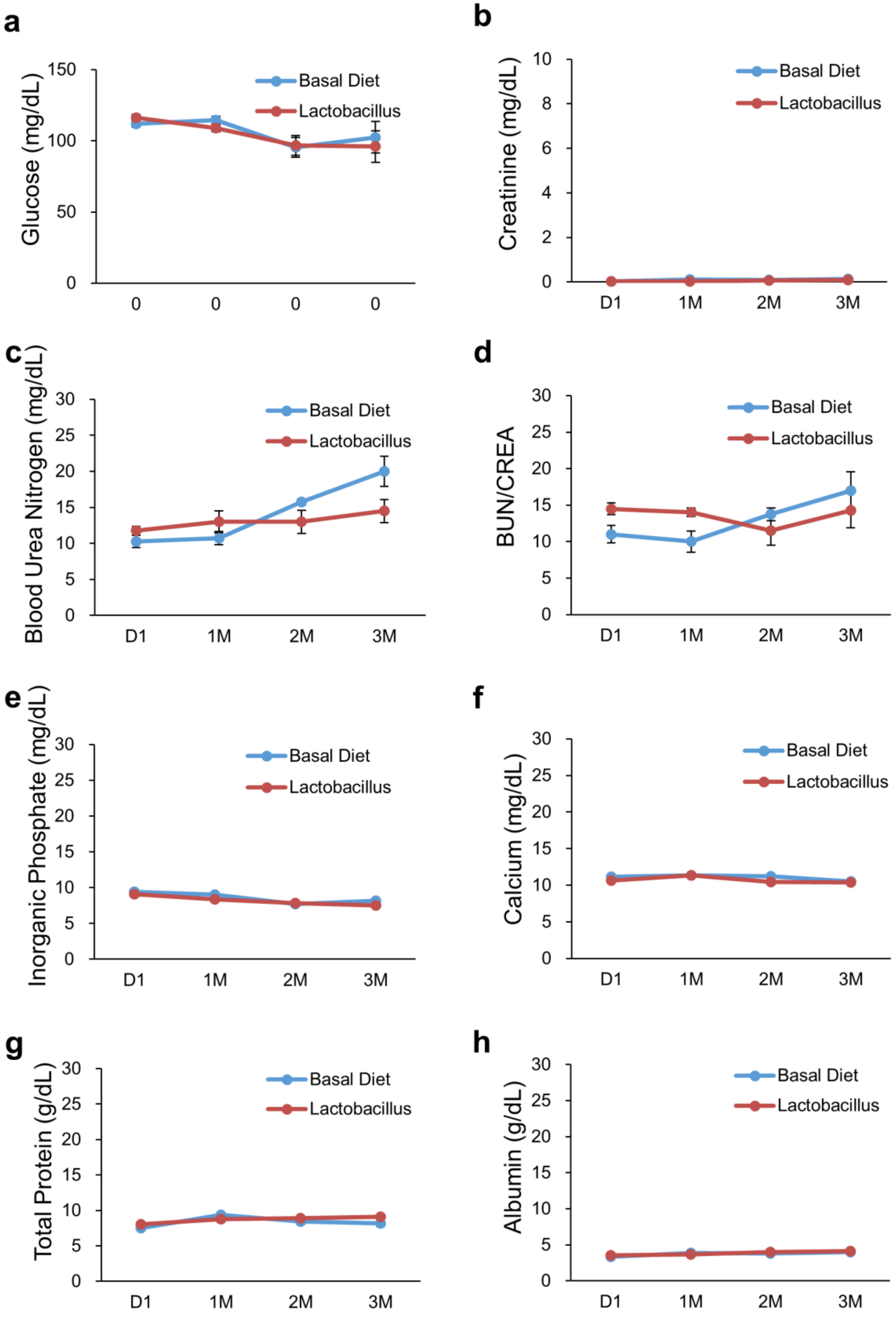

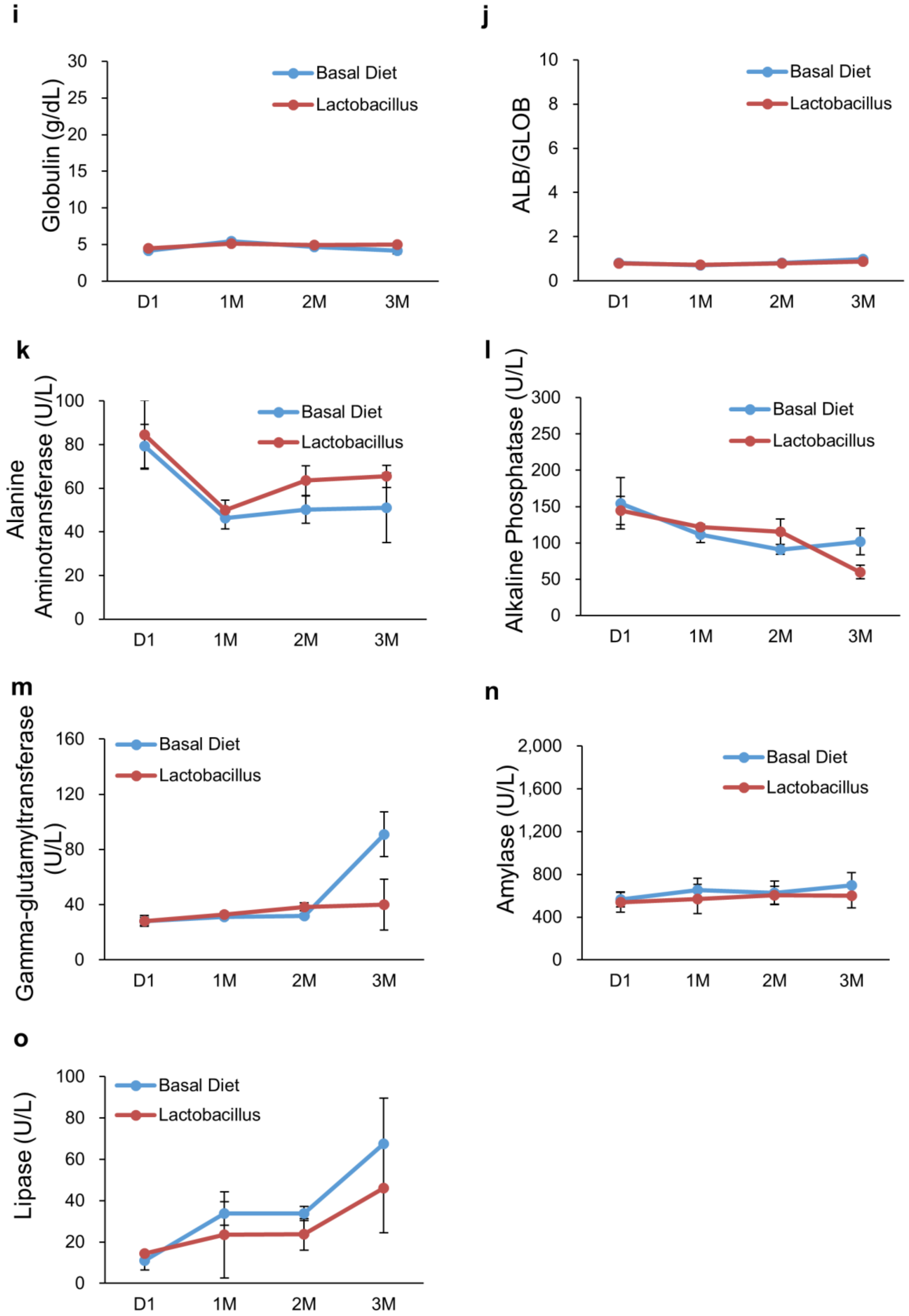
i
Globulin (g/dL)
30
25
20
15
10
5
0
D1
1M
2M
3M
Basal Diet
Lactobacillus
j
ALB/GLOB
10
8
6
4
2
0
D1
1M
2M
3M
Basal Diet
Lactobacillus
k
Alanine Aminotransferase (U/L)
100
80
60
40
20
0
D1
1M
2M
3M
Basal Diet
Lactobacillus
l
Alkaline Phosphatase (U/L)
300
250
200
150
100
50
0
D1
1M
2M
3M
Basal Diet
Lactobacillus
m
Gamma-glutamyltransferase (U/L)
160
120
80
40
0
D1
1M
2M
3M
Basal Diet
Lactobacillus
n
Amylase (U/L)
2,000
1,600
1,200
800
400
0
D1
1M
2M
3M
Basal Diet
Lactobacillus
o
Lipase (U/L)
100
80
60
40
20
0
D1
1M
2M
3M
Basal Diet
Lactobacillus

Supplementary Figure S1 Blood biochemical analysis of Jeju native pigs fed the multi-probiotic *Lactobacillus*. Whole-blood samples (n=9) were collected monthly during the experimental period, and blood samples were incubated at room temperature for 1 h and centrifuged at 1,700 × *g* for 30 min to separate the blood plasma. Comprehensive biochemistry profiles of blood plasma were analyzed on the same day of collection. (a) Glucose, (b) creatinine (CREA), (c) blood urea nitrogen (BUN), (d) BUN/CREA, (e) inorganic phosphate, (f) calcium, (g) total protein, (h) albumin (ALB), (i) globulin (GLOB), (j) ALB/ GLOB, (k) alanine aminotransferase, (l) alkaline phosphatase, (m) gamma-glutamyltransferase, (n) amylase, and (o) lipase.

Supplement Figure S2

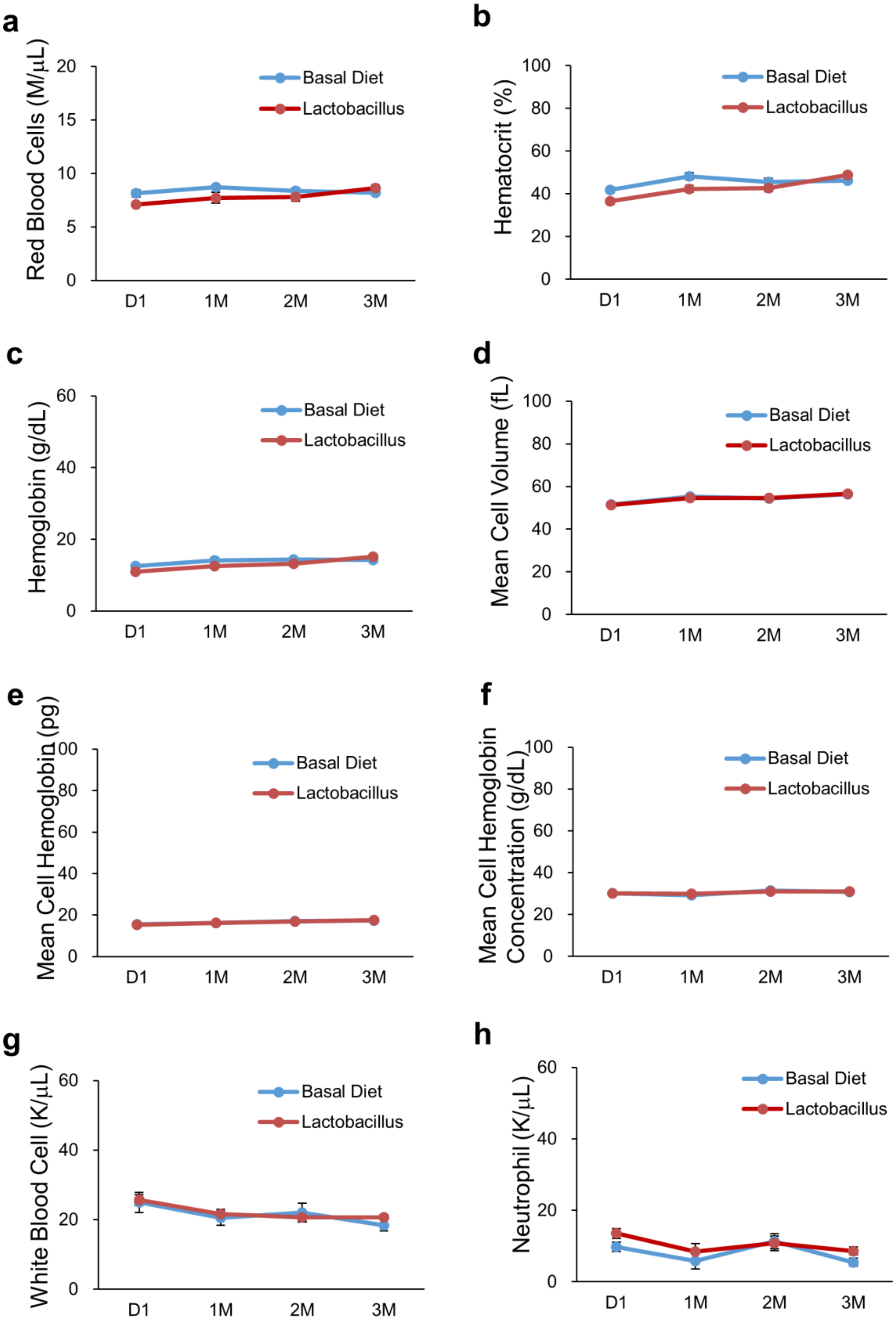

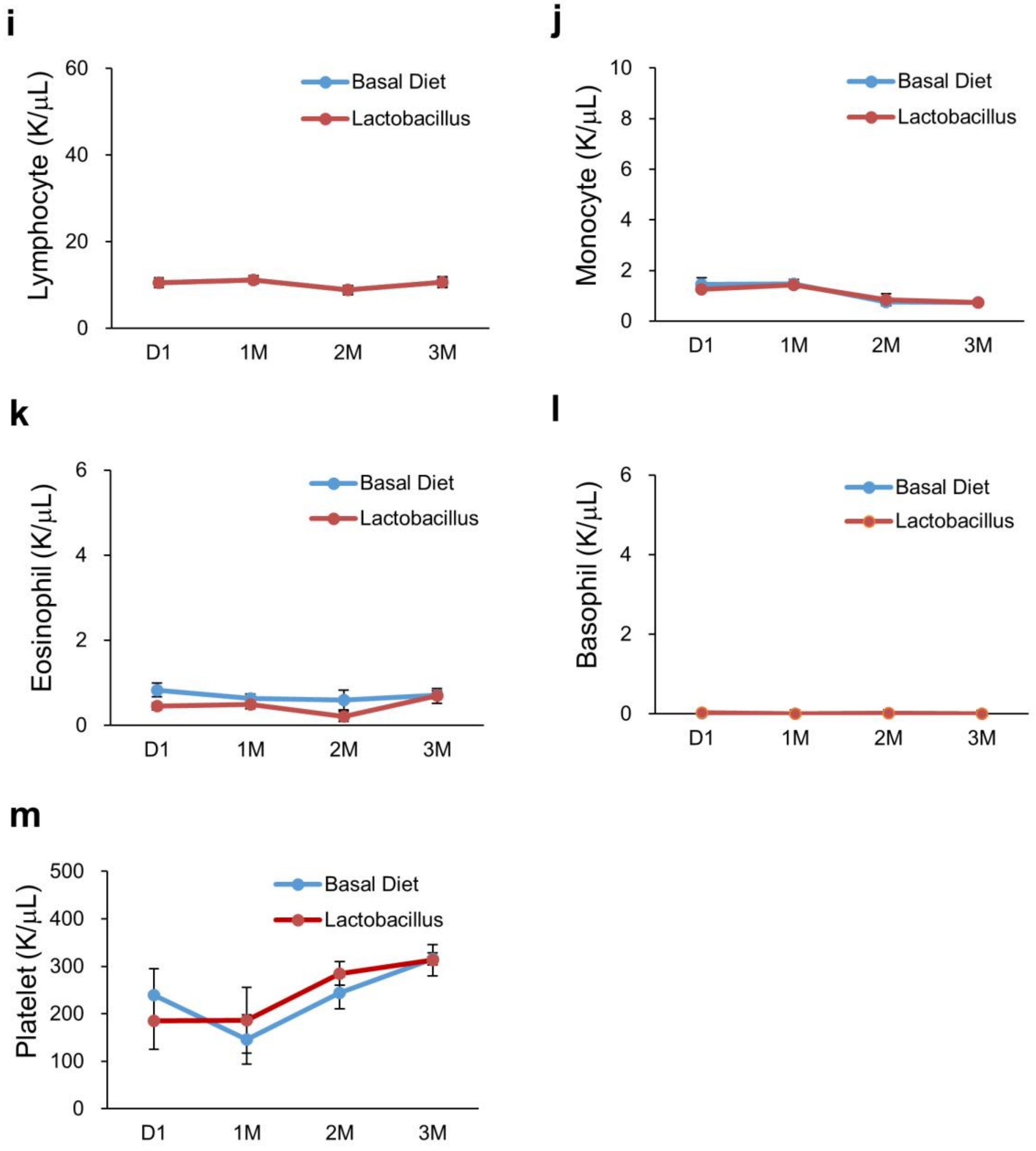

i
Lymphocyte (K/μL)
60
40
20
0
D1
1M
2M
3M
Basal Diet
Lactobacillus
j
Monocyte (K/μL)
10
8
6
4
2
0
D1
1M
2M
3M
Basal Diet
Lactobacillus
k
Eosinophil (K/μL)
6
4
2
0
D1
1M
2M
3M
Basal Diet
Lactobacillus
l
Basophil (K/μL)
6
4
2
0
D1
1M
2M
3M
Basal Diet
Lactobacillus
m
Platelet (K/μL)
500
400
300
200
100
0
D1
1M
2M
3M
Basal Diet
Lactobacillus

Supplementary Figure S2 Blood cell number profile of Jeju native pigs fed the multi-probiotic *Lactobacillus*. Whole-blood samples (n=9) were collected monthly during the experimental period, and the complete blood count was analyzed on the same day of collection. (a) Red blood cells, (b) hematocrit, (c) hemoglobin, (d) mean cell volume, (e) mean cell hemoglobin, (f) mean cell hemoglobin concentration, (g) white blood cell, (h) neutrophil, (i) lymphocyte, (j) monocyte, (k) eosinophil, (l) basophil, and (m) platelet.

Supplement Figure S3

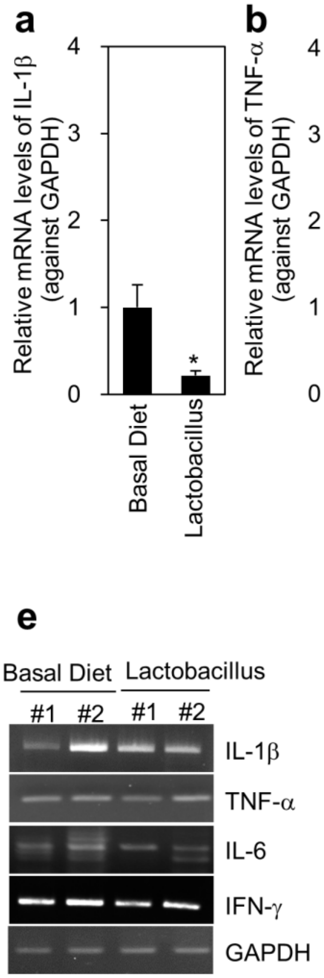

Supplementary Figure S3 Expression of immune-response related cytokines in the proximal colon tissue of Jeju native pigs fed the multi-probiotic *Lactobacillus*. RT-PCR was used to analyze the expression of the immune-response-related cytokines IL-1β, TNF-α, IL-6, and IFN-γ in the colon tissues of both diet groups. (a-d) qRT-PCR results of IL-1β, TNF-α, IL-6, and IFN-γ ($n = 9$). (e) Conventional RT-PCR results of IL-1β, TNF-α, IL-6, and IFN-γ in

experimental adipose tissues. The data are presented as the mean ± standard error of the mean, as analyzed by two-tailed Student's *t*-test. *$P < 0.05$, compared to the basal diet group.

Supplement Figure S4

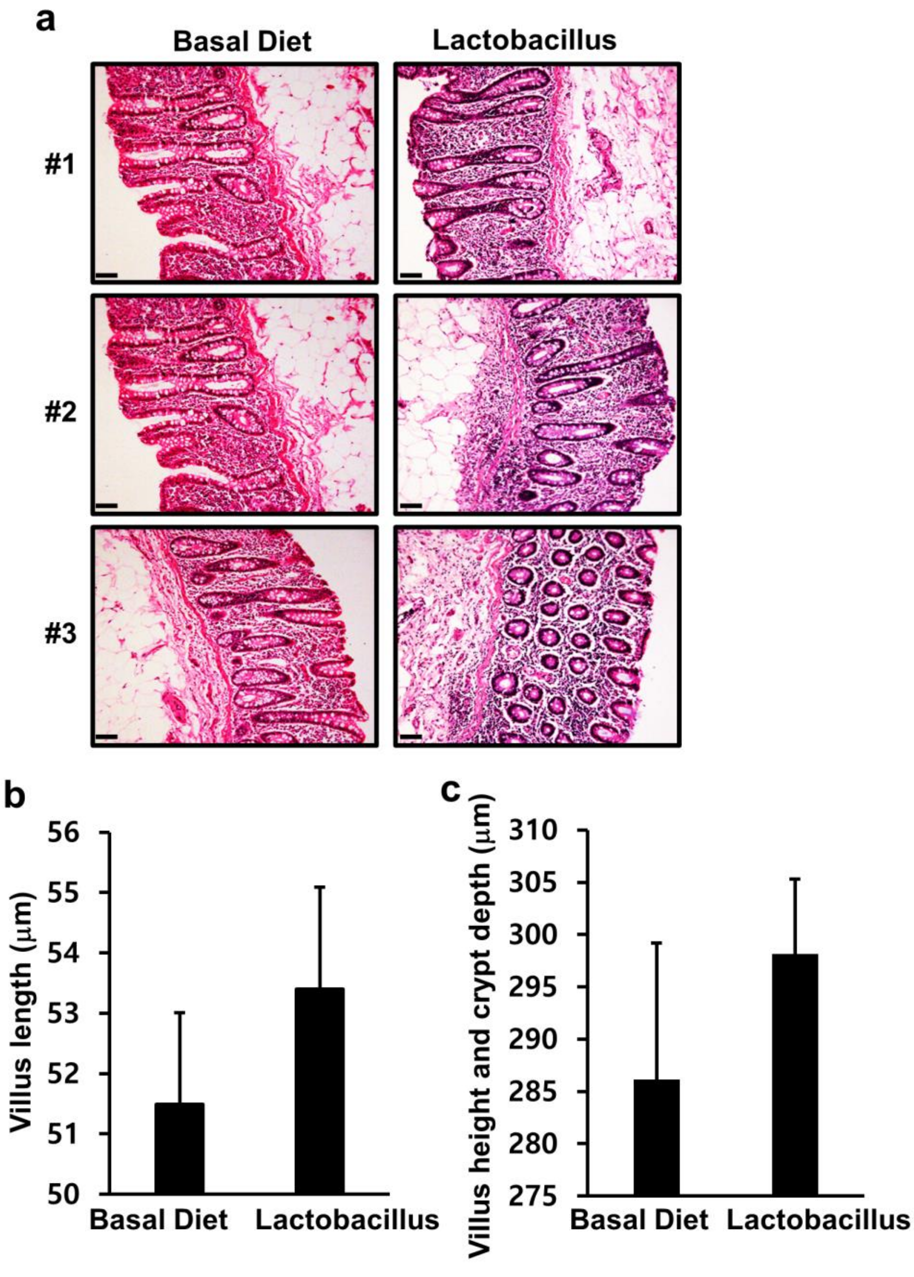

Scale bar: 50μm

Supplementary Figure S4 Histological analysis of the proximal colon tissue of Jeju native pigs fed the multi-probiotic *Lactobacillus*. Colon tissues were collected after the experimental period and preserved in 4 % paraformaldehyde. The tissues were then fixed and stained with hematoxylin and eosin. (a) Microscopic view of the hematoxylin and eosin-stained colon tissue. (b,c) Measured villus length and height in experimental pigs. The data are presented as the mean ± standard error of the mean, as analyzed by two-tailed Student's *t*-test (n =4). Scale bar: 50 μm

Supplement Figure S5

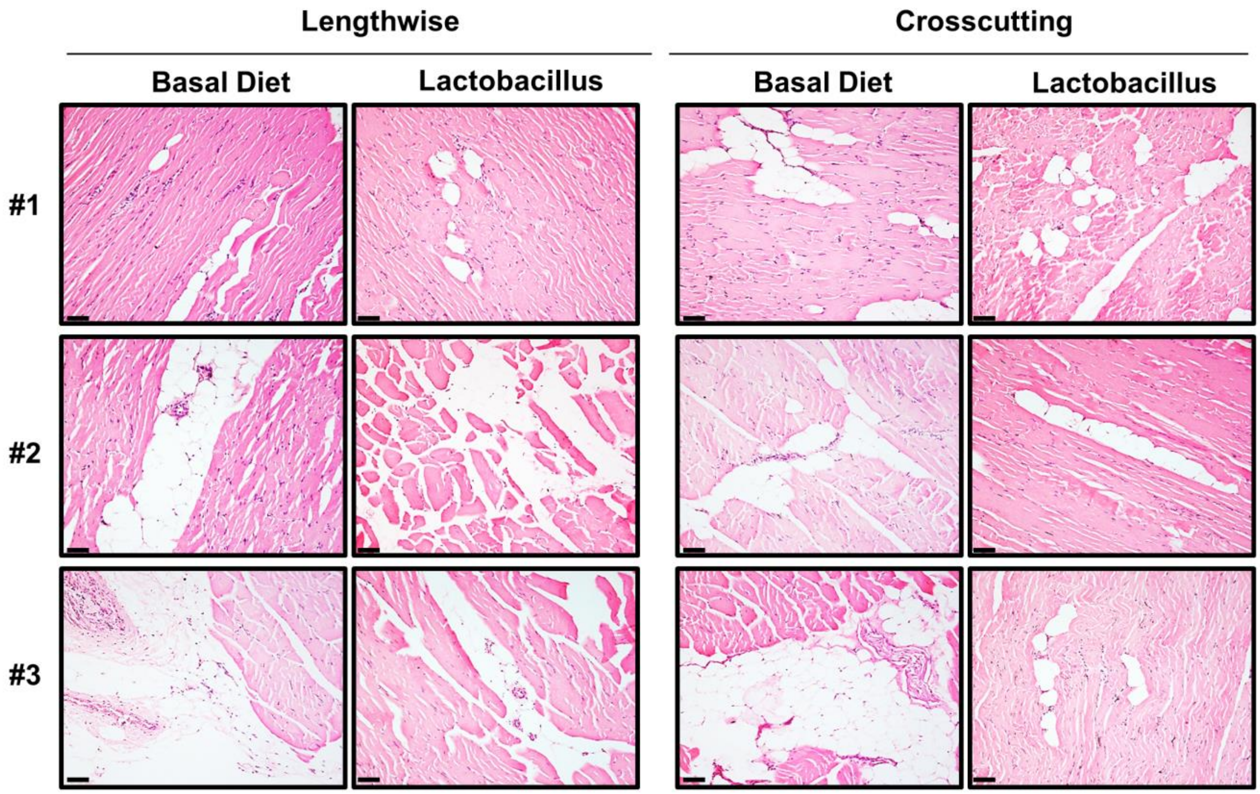

Supplementary Figure S5 Histological analysis of the *longissimus dorsi* muscle of Jeju native pigs fed the multi-probiotic *Lactobacillus*. Muscle tissues were collected after the experimental period and preserved in 4 % paraformaldehyde. Lengthwise and crosswise sections of the tissues were fixed and stained with hematoxylin and eosin (n =4). Scale bar: 50 μm

Supplement Figure S6

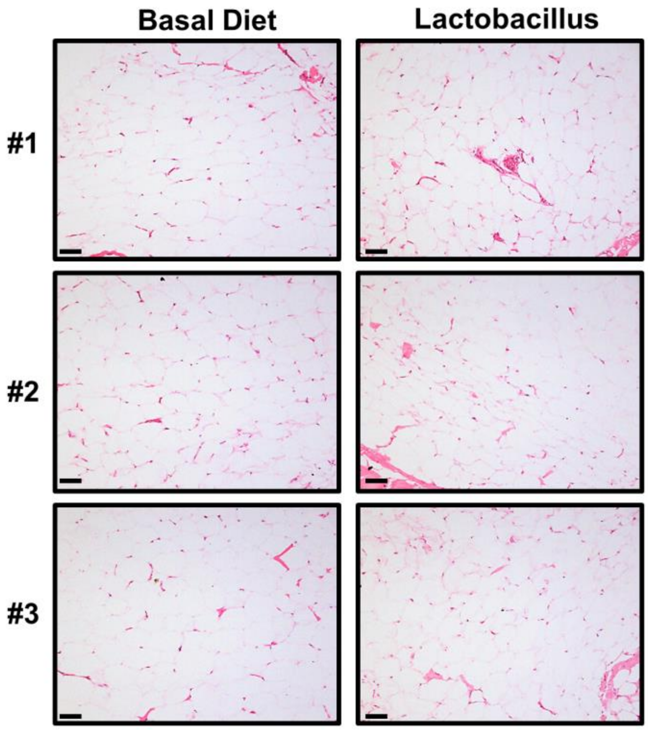

Supplementary Figure S6 Histological analysis of the adipose tissue of Jeju native pigs fed the multi-probiotic *Lactobacillus*. Adipose tissues were collected after the experimental period and preserved in 4 % paraformaldehyde. The tissues were then fixed and stained with hematoxylin and eosin (n=4). Scale bar: 50 μm

Supplement Figure S7

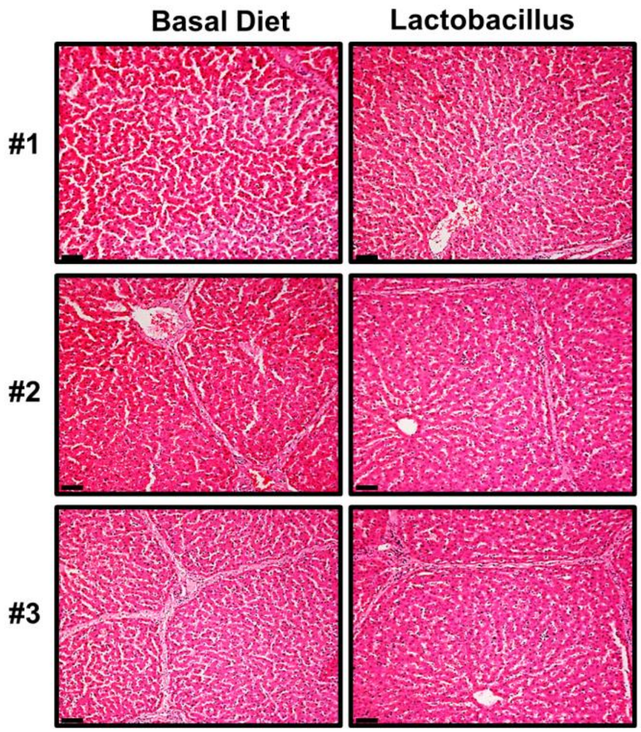

Supplementary Figure S7 Histological analysis of the liver tissue of Jeju native pigs fed the multi-probiotic *Lactobacillus*. Liver tissues were collected after the experimental period, preserved in 4 % paraformaldehyde, fixed, and stained with hematoxylin and eosin (n=4). Scale bar: 50 μm

Supplement Figure S8

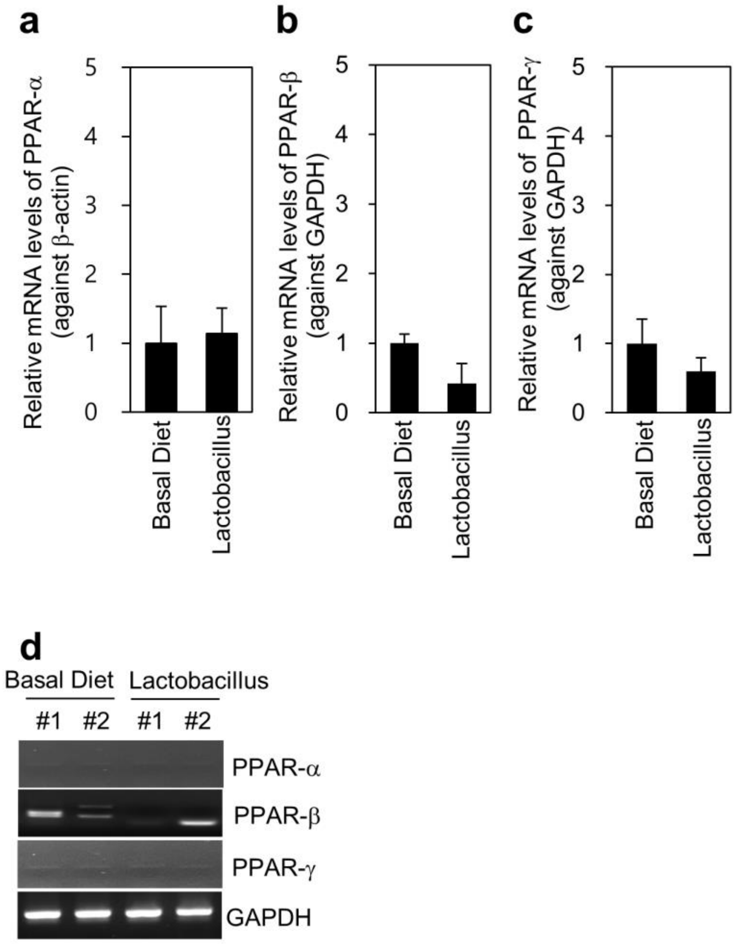

Supplementary Figure S8 Expression of PPAR family members in the adipocyte tissue of Jeju native pigs fed the multi-probiotic *Lactobacillus*. The expression of PPAR family members, including PPAR-α, PPAR-β, and PPAR-γ, in adipose tissues was analyzed by RT-PCR. (a-c) qRT-PCR results of PPAR-α, PPAR-β, and PPAR-γ (n = 9). (d) Conventional PCR results of PPAR-α, PPAR-β, and PPAR-γ in experimental adipose tissues. The data are presented as the mean ± standard error of the mean.

Supplement Figure S9

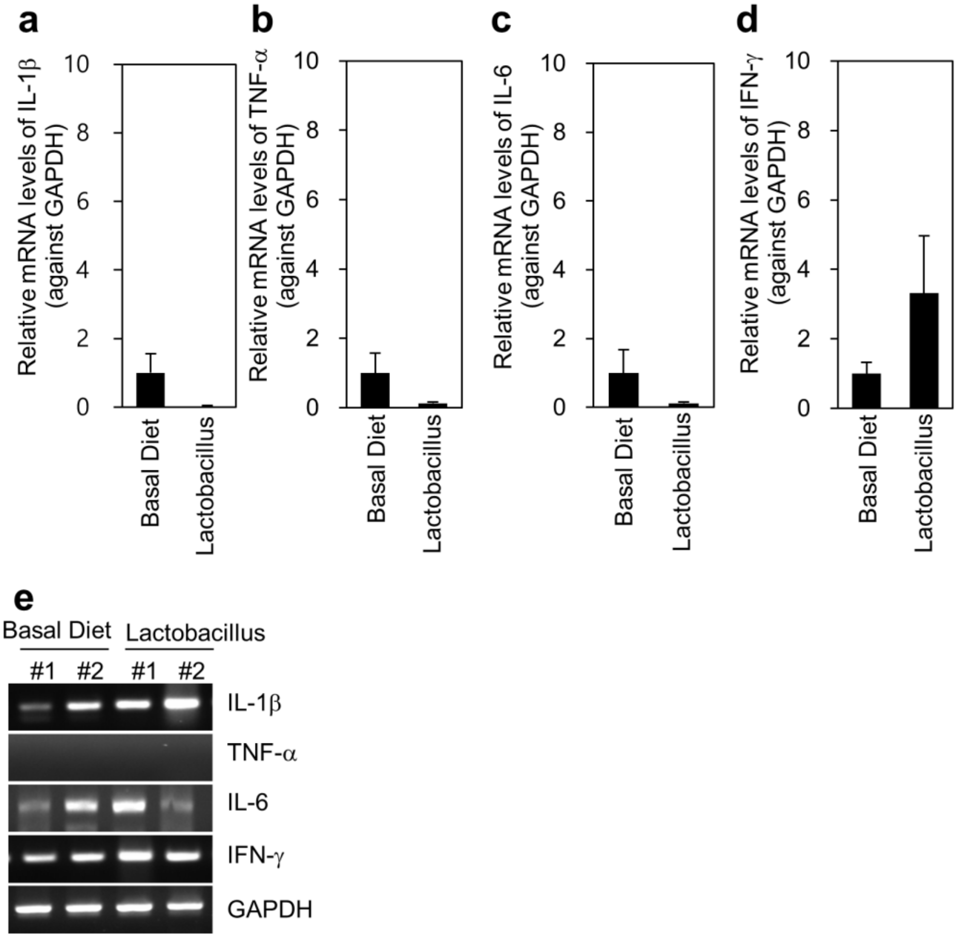

Supplementary Figure S9 Expression of immune-response related cytokines in the adipocyte tissue of Jeju native pigs fed the multi-probiotic *Lactobacillus*. RT-PCR was used to analyze the expression of the immune-response-related cytokines IL-1β, TNF-α, IL-6, and IFN-γ in the fat tissues of both diet groups. (a-d) qRT-PCR results of IL-1β, TNF-α, IL-6, and IFN-γ (n = 9). (e) Conventional RT-PCR results of IL-1β, TNF-α, IL-6, and IFN-γ in experimental adipose tissues. The data are presented as the mean ± standard error of the mean.

Supplement Figure S10-unclopped images related with Figure 2

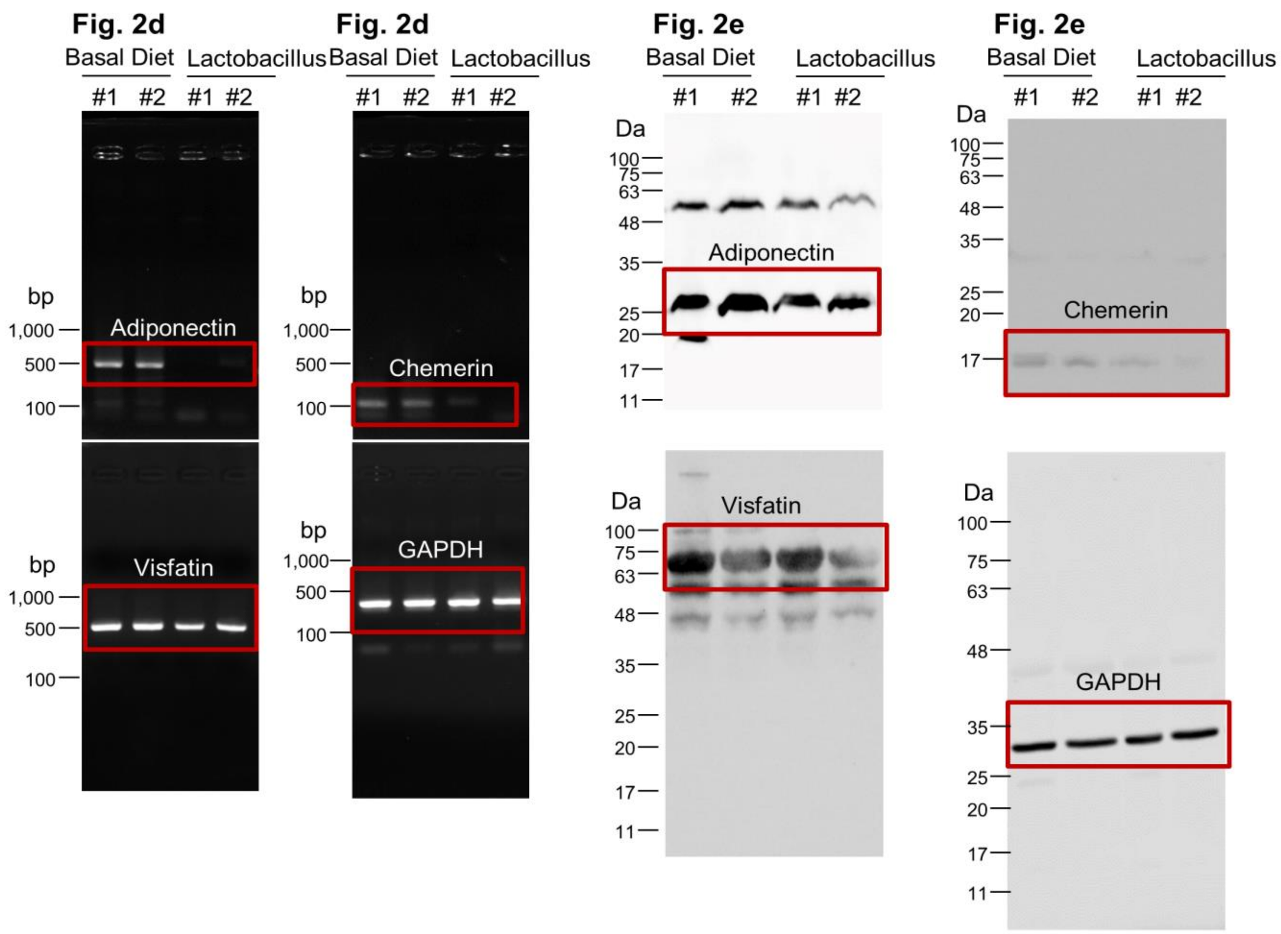

Supplement Figure S11-unclopped images related with Figure 3

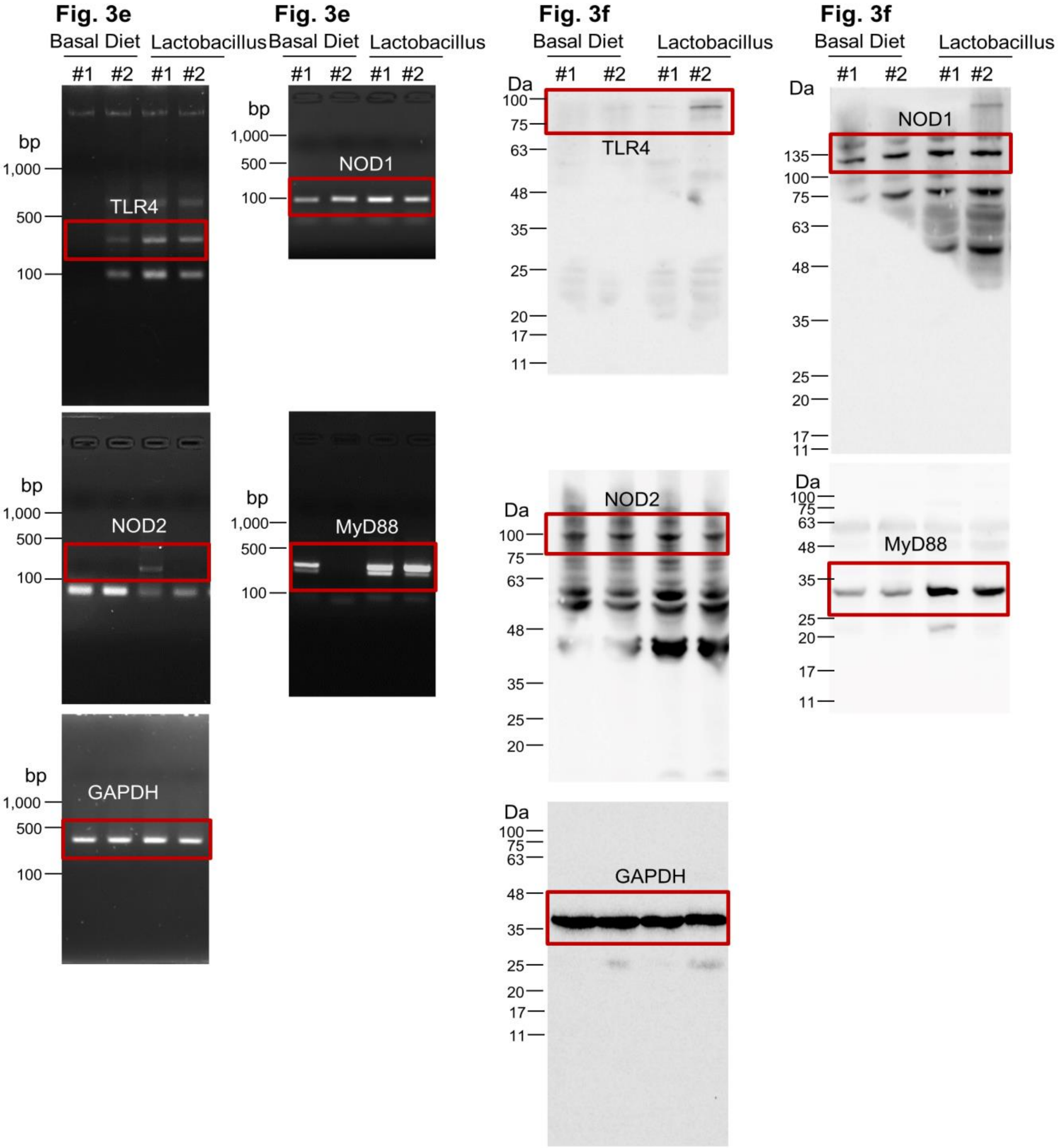

Supplement Figure S12-unclopped images related with Figure 4

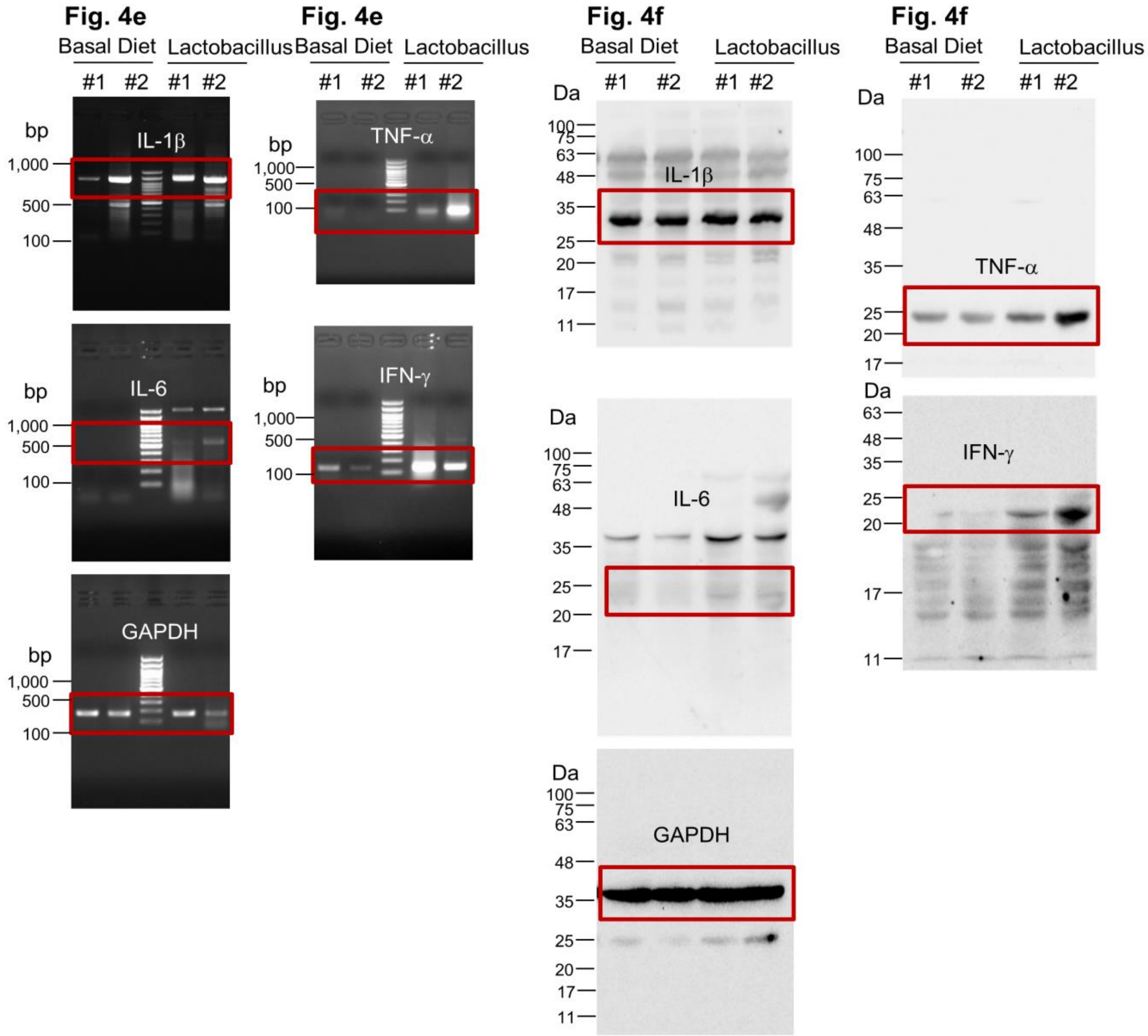

Supplement Figure S13-unclopped images related with Figure 5

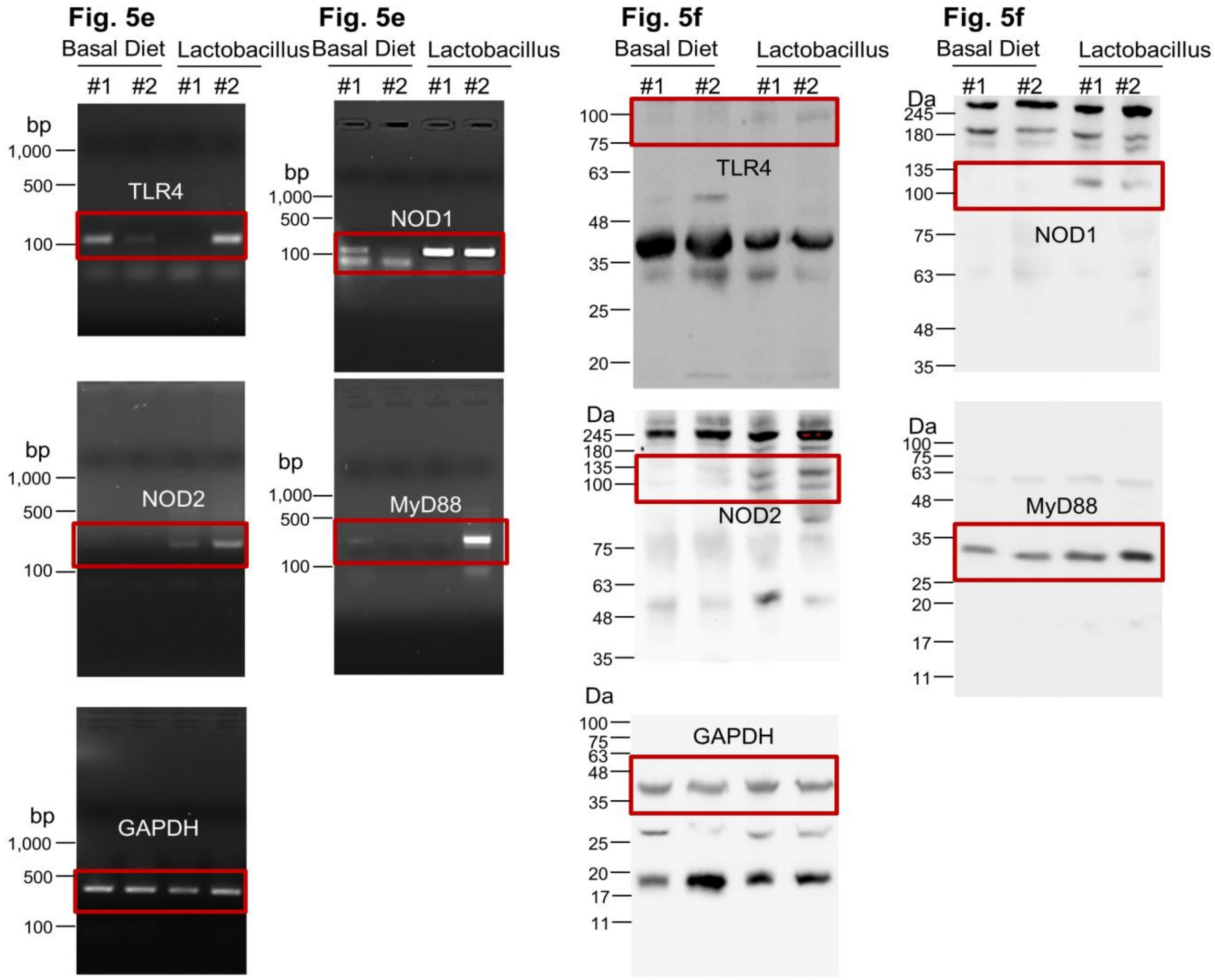

Supplement Figure S14-unclopped images related with Supplementary Figure S3e

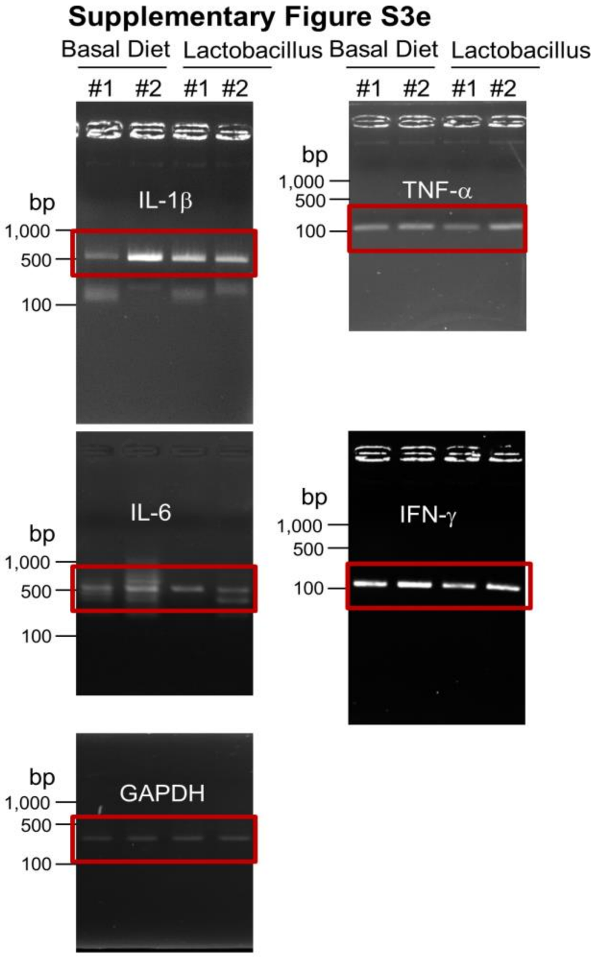

Supplement Figure S15-unclopped images related with Supplementary Figure S8d

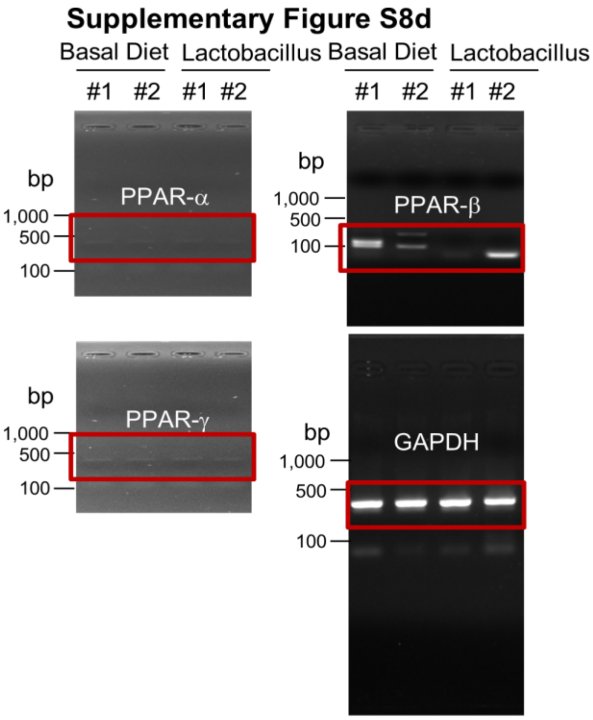

Supplement Figure S16-unclopped images related with Supplementary Figure S9e

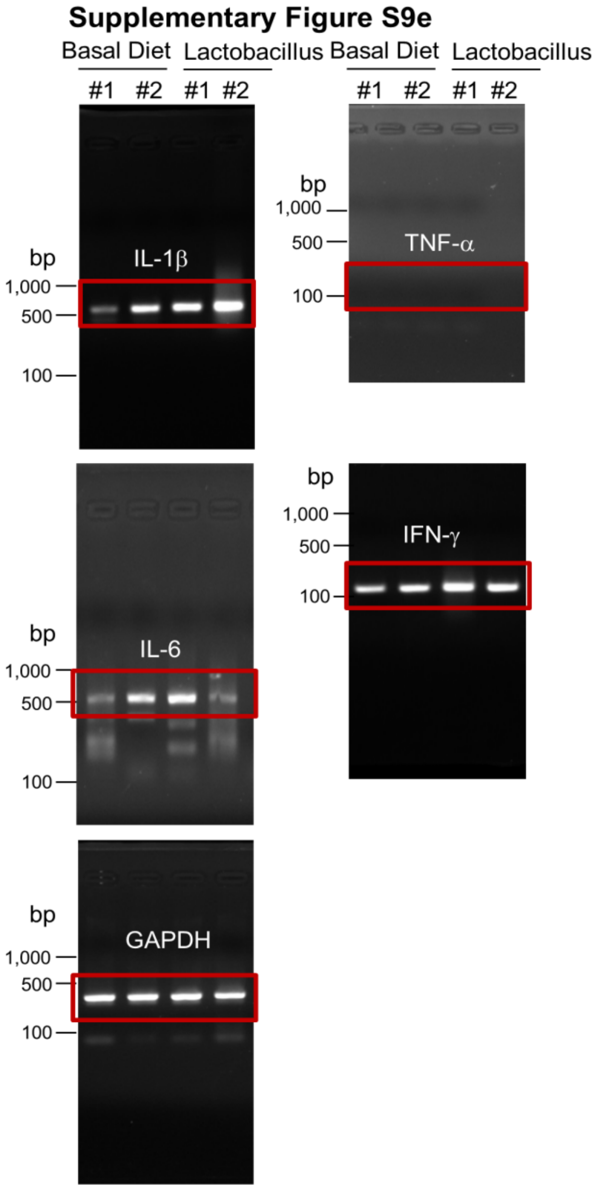

Supplement: Supplementary file 1 [file animals-11-02309-s001.zip › animals-1304749-supplementary.pdf]
